# A Pilot Study of Lung Clearance Index as a Useful Outcome Marker in the Follow-Up of Pediatric Patients with Non-Cystic Fibrosis Bronchiectasis?

**DOI:** 10.3390/children10050791

**Published:** 2023-04-27

**Authors:** Wael Alkoussa, Laurence Hanssens, Valerie Sputael, Frederico De Lucia, Christine Quentin

**Affiliations:** 1 Department of Pulmonology and Allergology, Queen Fabiola Children’s University Hospital, Université Libre de Bruxelles, 1020 Brussels, Belgium; laurence.hanssens@huderf.be (L.H.); valerie.sputael@huderf.be (V.S.); christine.quentin@huderf.be (C.Q.); 2 Department of Radiology, Queen Fabiola Children’s University Hospital, Université Libre de Bruxelles, 1020 Brussels, Belgium; fede.moli@live.it

**Keywords:** primary ciliary dyskinesia, multiple breath washout, spirometry, forced expiratory volume in one second

## Abstract

The forced expiratory volume in one second (FEV1) is regularly used for the follow-up of patients with non-cystic fibrosis bronchiectasis (nCF-BE). The lung clearance index (LCI), measured by the multiple breath washout test, has been recently proposed as a lung function measure and a potential tool more sensitive than the FEV1 measured by spirometry in assessing airway changes seen on imaging. While several data have been endorsed as a useful endpoint in clinical trials of patients with early or mild CF lung disease and as the main outcome measure in clinical trials with CFTR modulators in children and adolescents with CF, few data are available in the context of non-CF bronchiectasis. The aim of this pilot study was to compare the LCI with the FEV1 as well as the forced vital capacity (FVC), the forced expiratory flow at 25–75% of the FVC (FEF 25–75%), and chest imaging based on the modified Reiff score in patients with primary ciliary dyskinesia (PCD) and non-CF, non-PCD bronchiectasis (PCD-BE and nCFnPCD-BE). Additionally, we compared each test’s duration and the preferred technique. Twenty children were included; nine had PCD-BE and eleven had nCFnPCD-BE. The median age was twelve years (ages ranging between five and eighteen years). The median LCI was seven while the median z-scores of the FEV1, FVC, and FEF 25–75% were −0.6, 0, and −0.9, respectively. No significant associations or correlations were observed between LCI, spirometric parameters, or the modified Reiff score. However, nearly half of the population (*n* = 9) had an abnormal LCI, while only 10% had an abnormal FEV1. A total of 75% of children preferred MBW, despite it lasting five times longer than spirometry. In this paper, the authors suggest that LCI might be useful in a cohort of pediatric patients with PCD-BE and nCFnPCD-BE for detecting early lung function changes during their follow-up. Additionally, MBW seems to be preferred by patients. These data may encourage further studies on this topic.

## 1. Introduction

Pulmonologists face many challenges in the assessment and follow-up of the pulmonary status in multisystemic diseases such as cystic fibrosis (CF) and primary ciliary dyskinesia (PCD), most notably in the detection of airway changes that can occur long before the appearance of symptoms. During these “silent years”, irreversible damage and loss of lung function occur that might have been avoided with earlier diagnosis and preventive measures [1].

Bronchiectasis, characterized by a progressive and often irreversible bronchial dilatation caused by structural changes in the bronchial wall and chronic airway inflammation, is a common feature of CF and PCD. Excluding the latter two diseases, previous pneumonia and recurrent lower airway infections are the most common causes of pediatric bronchiectasis. Other risk factors include primary immune deficiencies, foreign body aspiration, and structural airway abnormalities such as bronchomalacia and congenital tracheobronchomegaly [2]. PCD, non-CF, and non-PCD bronchiectasis (PCD-BE and nCFnPCD-BE) can be quite debilitating and lead to chronic respiratory symptoms such as recurrent cough, increased sputum production, and recurrent chest infections [3]. High-resolution computerized tomography (CT) remains the gold standard for the diagnosis of PCD-BE and nCFnPCD-BE. Management of bronchiectasis is multidisciplinary and mainly focused on reducing exacerbations. The mainstays of treatment are antibiotics and airway clearance techniques, which aim to maintain or improve both lung function and quality of life [2,3].

Forced expiratory volume in one second (FEV1) is widely used in the follow-up of PCD-BE and nCFnPCD-BE because it is readily available in pulmonology departments and its reproducibility is well established. However, there is increasing evidence that FEV1, the reference parameter for the diagnosis and follow-up of obstructive lung diseases, is unable to detect early functional lung impairment as well as structural airway changes and their progression in these patients [3,4].

One of the techniques that can be used is the multiple breath washout (MBW). While FEV_1_ primarily reflects central airway obstruction, lung clearance index (LCI), the most commonly reported parameter of MBW, reflects global ventilation heterogeneity by quantifying uneven ventilation distribution [5,6]. LCI is defined as the cumulative expired volume at the point where end-tidal inert gas concentration falls below 1/40th of the original concentration divided by the functional residual capacity [6]. Due to the higher volume of the lung periphery compared to the central airways, LCI reflects peripheral lung function more than FEV1 or other parameters derived from spirometry. Spirometry is, in fact, limited in its ability because it is dependent on the airway resistance, which is very low in the airways starting at the eighth to tenth generation. MBW has been proven to be useful in patients with CF, but its place in the follow-up of patients with PCD-BE and nCFnPCD-BE remains unclear [7]. Studies conducted on MBW in PCD-BE patients are conflicting and often not comparable because of differences in MBW devices, tracer gases, and the equipment being used [8]. While LCI could potentially be more sensitive than FEV1 in detecting early and mild lung disease in children with PCD-BE, it has already been shown to be a sensitive tool in adults with nCFnPCD-BE, which leaves more work to be done in this field concerning these two entities [8,9].

The main objective of this pilot study was to compare LCI and FEV1 values for detecting lung function abnormalities in children with PCD-BE and nCFnPCD-BE. Another objective was to compare LCI with other spirometric parameters such as forced expiratory flow at 25–75% (FEF 25–75%) and forced vital capacity (FVC), as well as with the modified Reiff score on a chest computed tomography (CT) scan. We also compared the duration of each test (MBW vs. spirometry) and assessed patients’ preferred technique according to a satisfaction survey sheet.

## 2. Materials and Methods

### 2.1. Study Design

This is a single-center, prospective, observational study concerning patients with PCD-BE and nCFnPCD-BE followed at Queen Fabiola Children’s University Hospital (HUDERF) of the Université Libre de Bruxelles (ULB), Belgium. Participants were recruited over 6 months between May and October of 2021.

### 2.2. Study Population

This study targeted children aged between 5 and 18 years who were capable of understanding and performing MBW and spirometry. These children were known to have either nCFnPCD-BE confirmed on CT scan according to specific criteria (internal diameter of a bronchus is wider than its adjacent pulmonary artery with a broncho-arterial ratio >0.8, failure of the bronchus to reduce in thickness, and visualization of the bronchi in the outer 1–2 cm of the lung fields) [2,10] or to have PCD-BE. PCD was diagnosed either by ciliary biopsy, studying ultrastructure through electron microscopy, by ciliary beat pattern through digital high-speed video microscopy, or by genetic testing [11]. These patients are scheduled if exacerbations occur. All patients had been diagnosed more than 6 months prior to enrollment and were clinically stable at the time of testing.

Patients less than 5 years old or more than 18 years old were excluded from the study. Patients who were known to have CF or who had any exacerbations requiring systemic antibiotics within the week prior to the visit were also excluded. Additionally, patients who could not perform spirometry or MBW were excluded, as were patients with a history of surgical lobectomy.

All patients with PCD and non-CF bronchiectasis who fit the specified inclusion criteria were selected during a routine follow-up visit and were invited to participate in the study [10]. The study started after obtaining the informed consent of the patients and/or their parents.

### 2.3. Collected Data

Patients’ demographic characteristics included age, sex, ethnicity, and BMI Z-scores, which were adapted to ethnicity. These were collected along with the type of pathology, spirometric parameters (FEV1, FVC, and FEF 25–75%), and MBW (LCI).

For radiological assessment, the modified Reiff score is frequently used. It incorporates radiological findings of bronchiectasis in the six lobes of the lungs, with the lingula being counted as a separate lobe. The scoring of bronchial dilatation is quantified relative to the adjacent pulmonary arteries as follows: 0 (none, normal), 1 (100–200% arterial diameter), 2 (200–300% arterial diameter), 3 (>300% arterial diameter), and the maximum score is 18 [12]. Bronchiectasis is defined as bronchial dilatation above 100% of the adjacent pulmonary arteries in adults, while in children, this percentage drops to 80%. To apply the score to our pediatric population and stay consistent with the definition of bronchiectasis in children, we gave a normal score of 0 when the bronchial dilatation compared to adjacent pulmonary arteries was between 0 and 80% and a score of 1 when the range was 80–200%. The percentage ranges used for scores of 2 and 3 remained the same as in the initial definition. A qualified radiologist with expertise in pediatric pulmonology and bronchiectasis applied the modified Reiff score to the most recently performed CT scan available for each participant. No CT scans were performed as part of the study in order to prevent unnecessary radiation exposure.

### 2.4. Procedures

**Figure 1:**
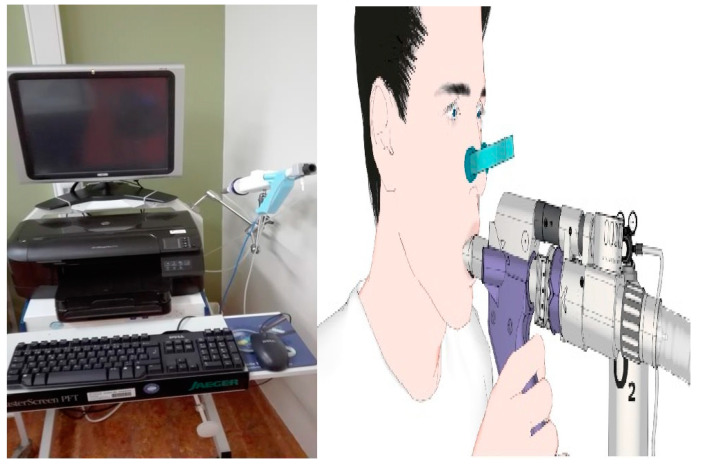
The machine for the multiple breath washout and the technique used during the test.

MBW: Patients performed an MBW test using nitrogen (N_2_) on a scheduled routine visit, followed by spirometry. The nitrogen MBW (N_2_MBW) was performed using the EXHALYZER D technique and analyzed by SPIROWARE 3.3.1 software, consistent with the European Respiratory Society/American Thoracic Society consensus statement [13]. For each patient, a disposable pulmonary function filter was used and attached to a mouthpiece that was age-adapted. A nose clip was applied as well (Figure 1).

**Figure 2:**
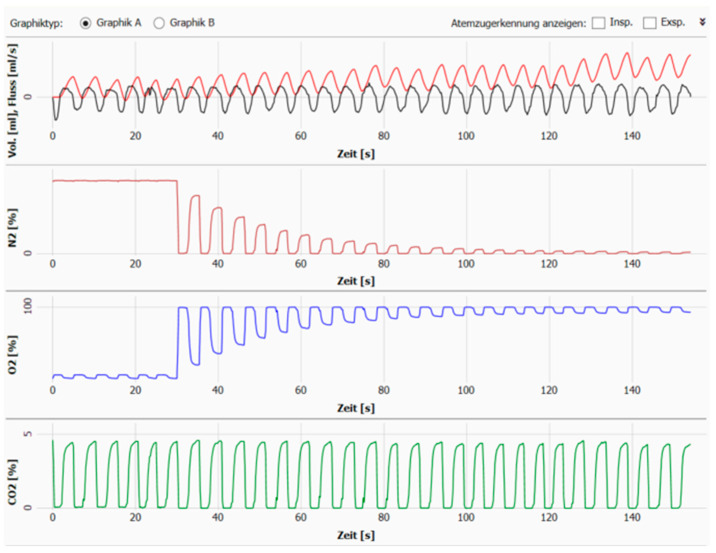
Example of the curves of N_2_, O_2_, and CO_2_ during a LCI measurement.

The mean values of the N_2_ MBW indices from three technically acceptable measurements were reported as absolute values. A measurement was considered acceptable when the three curves showing the evolution of N_2_, O_2_,and CO_2_ were completely homogenous with a proportional evolution and without any leaks or forceful respirations (Figure 2). If a measurement was not acceptable, an additional measurement was performed to replace it.

LCI is the index of overall ventilation inhomogeneity evaluated in the study. An index of 7.1 is considered to be the upper limit of normal for healthy subjects older than 5 years. In our study, any LCI > 7.1 was considered abnormal [14].

The testing was performed by a single pediatric pulmonologist in an office isolated from surrounding noise. Children were allowed to watch a movie of their choice on a tablet and were asked to sit as still as possible during the testing. A stopwatch was used to record the duration required for all three trials of the testing to be done, including breaks between two consecutive trials.

Spirometry: Dynamic lung volumes were measured by spirometry according to European Respiratory Society/American Thoracic Society standards [15]. Z-scores were calculated from the Global Lung Function Initiative [16]. Normal values were considered to be between −1.6 and +1.6. Spirometric values were considered abnormal if the z-scores were outside of this range [15,16,17].

**Figure 3:**
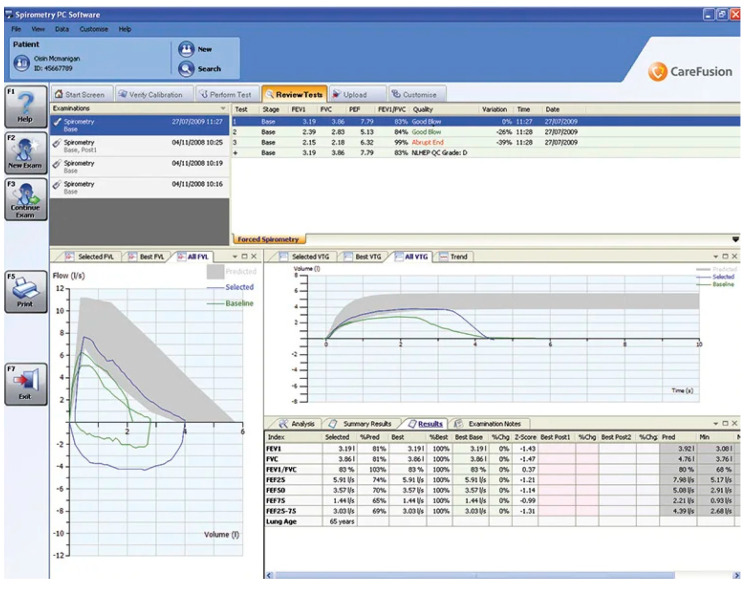
Example of spirometric measurement curves.

The test was conducted by an experienced registered nurse at a pulmonology testing laboratory where the children had previously performed tests during follow-up visits (Figure 3). Children also had the option to play a challenging game during the test. A stopwatch was used to record the duration of testing.

After completion of both tests, a satisfaction survey sheet about which technique was preferred was given to each child to be filled out in the presence of their parents. This survey consisted of 5 questions asking which technique children considered to be the easiest, the most comfortable, the fastest, and the most entertaining. The last question asked children which technique they preferred to have repeated at the next visit. Each answer was granted 1 point, and the child’s overall preferred technique was considered to be the one that tallied 3 or more points.

### 2.5. Statistical Analysis

Baseline population characteristics were presented as median (range) or frequency (percentage), when appropriate.

The primary purpose of the statistical analysis was to assess and compare global and individual differences between LCI and FEV1 values. Correlation tests were also used to compare LCI with the different parameters of spirometry and the modified Reiff score using the Pearson’s R correlation test, with a *p*-value less than 0.05 and an R ratio close to +1 or −1 considered to be significant. To investigate all of our objectives, associations were analyzed using the Fisher’s exact test, the Mann-Whitney test, and the Wilcoxon test between variables of interest, with a *p*-value less than 0.05 considered to be significant.

Statistical analysis and graphs were done using GraphPad Prism 9.5.0 (GraphPad Software Inc., San Diego, CA, USA) and Microsoft Office Word and Excel (Microsoft Corporation, 2016, Impressa Systems, Santa Rosa, CA, USA).

This study was approved by the ethical committees of HUDERF and an academic hospital (ERASME hospital) under the codes 54/21 and P2021/254, respectively.

## 3. Results

Among thirty patients with PCD-BE and nCFnPCD-BE who presented to the pulmonology unit of HUDERF during the study period, twenty were enrolled. Seven patients chose not to enroll in the study for the following reasons: lack of free time to participate in a study (*n* = 2), parental concerns regarding a new technique (*n* = 2), and patients’ fear of the new machine used to perform MBW (*n* = 3). Three patients were excluded because of a concomitant psychiatric condition, an ongoing acute exacerbation in a PCD patient, and a history of total pneumonectomy. Aside from the patient excluded due to an ongoing exacerbation, the patients were all stable and had not suffered any acute exacerbations in the preceding six months; this low rate of exacerbations was hypothesized to be a result of the lockdown measures taken by the government in response to the COVID-19 epidemic.

### 3.1. Baseline Demographic Features

Baseline demographic features are shown in Table 1. Participants’ age range was between five and eighteen years, with a median of twelve years. A total of 55% were males, 90% of participants were of Caucasian ethnicity, with two participants (10%) being persons of color. BMI ranged between a z-score of −1.3 and 2.3, with a median of 0.7.

### 3.2. Etiologies and Pulmonary Status

Etiologies: In patients with nCFnPCD-BE (*n* = 11), the identified etiology of bronchiectasis was recurrent pneumonia (64%), middle lobe syndrome (18%), Bruton’s agammaglobulinemia, and esophageal atresia.

Nine out of these eleven patients were diagnosed with bronchiectasis at the age of five years or older, while the remaining two patients were younger than five years when diagnosed with bronchiectasis.

Of the nine participants with PCD-BE, four were diagnosed due to the total absence of dynein arms on electron microscopy, four had abnormal ciliary movement, and one was diagnosed by genetic testing. All of these patients were found to have bronchiectasis at the age of five years or older.

Pulmonary status: Pulmonary status was assessed by LCI, FEV1, FVC, FEF 25–75%, and the Reiff score applied to chest CT scans of participants. The data are shown in Table 2, along with median values.

For the total population, the median LCI was seven, while FEV1, FVC, and FEF 25–75% had medians of −0.6, 0, and −0.9, respectively, in the z-score. We noted that only 55% of the participants had a normal LCI (33.3% of the patients with PCD-BE vs. 72.7% of the patients with nCFnPCD-BE), compared to 90% who had a normal FEV1 (all the patients with PCD-BE vs. 81.8% of patients with nCFnPCD-BE), 95% who had a normal FVC (all the patients with PCD-BE vs. 90.9% of patients with nCFnPCD-BE), and 85% who had a normal FEF 25–75% z-score (77.8% of patients with PCD-BE vs. 90.9% of patients with nCFnPCD-BE).

**Figure 4:**
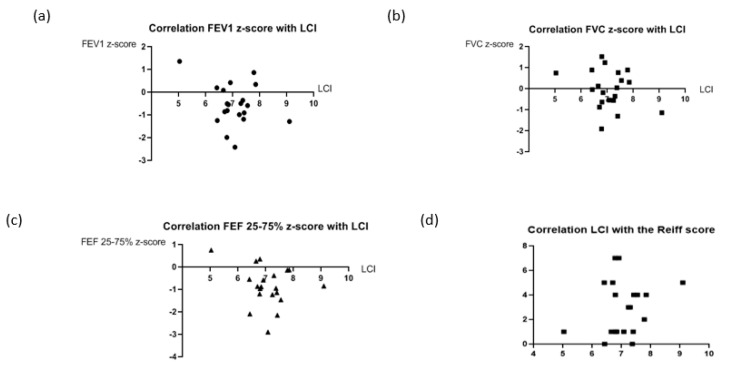
Correlations between the different spirometric parameters, the Reiff score, and the LCI. (**a**) non-significant correlation between LCI and FEV1 z-score (*n* = 20; R = −0.096 with a *p*-value of 0.34); (**b**) non-significant correlation between LCI and FVC z-score (*n* = 20; R = −0.07 with a *p*-value of 0.38); (**c**) non-significant correlation between LCI and FEF25-75% z-score (*n* = 20; R = −0.18 with a *p*-value of 0.22); (**d**) non-significant correlation between LCI and the Reiff score (*n* = 20; R = 0.117 with a *p*-value of 0.31).

After applying Fischer’s exact test to different possible combinations between LCI and the parameters of spirometry, there weren’t any significant associations, as shown in Table 3. Additionally, while applying the Pearson’s R correlation test, there weren’t any significant correlations between LCI and spirometric parameters (Figure 4). Alternatively, the Mann-Whitney test and the Wilcoxon test were used to find associations while comparing the medians of LCI, FEV1, FVC, and FEF of 25–75% of participants with PCD-BE to those with nCFnPCD-BE. Similarly, no significant associations were found between the different lung function parameters (*p*-values = 0.46; 0.8; 0.8 and 0.2, respectively).

The modified Reiff score applied to bronchiectasis found on chest CT scans of our 20 participants ranged between one and seven, with a median score of three, as presented in Table 3. Most of the CT scans (85%) were considered relatively recent, having been done within five years of this study (median: three years), while 15% of CT scans were more than five years old. No correlation was proven to exist between LCI and the modified Reiff score when applying the Pearson’s R correlation test (*p*-value = 0.31).

### 3.3. Satisfaction Survey

The median duration of each test was 18.5 min (15–25.6) for MBW and 4.3 min (2.3–8.7) for spirometry, meaning the duration of MBW was nearly five times that of spirometry.

After finishing the two tests, all participants were handed the satisfaction survey sheet.

A total of 80% of participants found MBW to be easier and more comfortable than spirometry, and 60% considered MBW to be more entertaining. All participants experienced spirometry as being faster than MBW. Most participants (75%) selected MBW over spirometry when asked which method they preferred for follow-up testing of their lung function.

## 4. Discussion

At the present time, few studies have been conducted to assess the usefulness of MBW in the follow-up of non-CF bronchiectasis. In our study, 9 participants out of 20 had an abnormal LCI, while 18 had a normal FEV1, 19 had a normal FVC, and 17 had a normal FEF 25–75% in terms of z-score.

Agreeing on an upper limit of normal for LCI was challenging. Based on a study conducted in different hospitals in the United Kingdom, LCI was found to have a wider range of normal results for adults than for children [7]. In addition, multiple MBW devices exist at present, and the values of LCI are not interchangeable. They are specific to the device, the software, and the tracer gas being used. In our study, the value of 7.1 was set as the upper limit of normal for healthy subjects aged more than five years [14]. Conventional spirometry changes with age, height, and gender, and its result is expressed as a percent predicted value or as a z-score. LCI is affected to a lesser degree by height, and this variation is unremarkable in the age range of our studied population. Furthermore, gender has no effect on LCI [1].

When comparing LCI to the different parameters of spirometry, no significant associations were found, suggesting that LCI cannot be predicted by the parameters of spirometry, and likewise, the parameters of spirometry cannot be predicted by LCI. While LCI can be completely abnormal, spirometry parameters may still be normal. This suggests that LCI might be useful as an earlier marker of pulmonary function changes in participants with PCD-BE or with nCFnPCD-BE.

Although there weren’t any significant correlations between LCI and the spirometric parameters, we observed that the median of LCI (7) was close to the upper limit of normal compared to the median of FEV1 (−0.6 in the z-score), which was well within the normal range. A similar observation was seen when comparing LCI to FVC and to FEF 25–75%. Similar trends were described in two cross-sectional studies conducted at a national center for PCD in Copenhagen, Denmark [4]. Controversially, in a longitudinal study conducted at the same center in 2019, which followed the evolution of LCI in PCD patients over the course of one year, LCI significantly increased in participants, and a moderate negative correlation between LCI and FEV1 was shown. This correlation highlights the impact of the limited number of participants in both groups of our study [4]. Moreover, two patients from our study population with nCFnPCD-BE were found to have abnormal FEV1 in the z-score with a normal LCI. When excluding these two patients, the observation mentioned earlier applies, with nine out of eighteen children having normal spirometry and an abnormal LCI (six children out of nine with PCD-BE and three out of nine with nCFnPCD-BE). Even though it is known that LCI might be elevated in patients with PCD in the setting of impaired mucociliary clearance and yet absent bronchiectasis, one third of our patients with PCD-BE had a normal LCI, suggesting that PCD by itself does not always cause elevated LCI. In conclusion, our study suggests that MBW might be a sensitive tool for detecting peripheral airway changes earlier than spirometry, with more confirmatory studies needing to be carried out with larger patient populations as well as with comparisons of LCI between patients and healthy subjects [4].

Despite applying a modified Reiff score to our population, as previously explained, there was no significant correlation observed between this radiological score and LCI. Thus, the number of lobes affected by bronchiectasis does not seem to correlate with LCI abnormalities. While CT scans remain the gold standard for the diagnosis and evaluation of bronchiectasis as well as for the follow-up of CF and PCD, use of this technique should be limited to an absolute minimum given the substantial associated dose of radiation. Our study was thus limited by the inability to perform CT scans of patients at the same timepoint as when LCI was performed. Hence, the comparison between LCI and CT findings presented above is only a preliminary one, and more investigations are needed. While magnetic resonance imaging (MRI) may be more helpful and informative than CT and does not expose the patient to ionizing radiation, it unfortunately has less sensitivity in detecting peripheral bronchiectasis [2]. Both modalities require the sedation of young children in order to obtain high-quality images. In contrast, the MBW test is a non-invasive functional measurement with minimal risks or hazardous exposures. MBW may also be preferred over imaging based on the fact that lung function changes are not directly related to the severity of the imaging findings. For the above reasons, we believe that MBW might be useful in the long-term follow-up of patients with non-CF bronchiectasis [18,19]. While it remains to be seen whether LCI is a sensitive method for detecting early structural changes in pediatric lung diseases, it is not able to distinguish between reversible and irreversible changes. In this regard, MRI remains important as a sensitive tool for monitoring lung disease progression without radiation exposure [20].

Despite our findings that MBW (which requires a wash-in and washout phase with each trial lasting approximately five min and three trials needed overall) took nearly five times longer than spirometry, 75% of the participants indicated preference for MBW over spirometry [1]. This might be attributed to the fact that children had the option to watch a program of their choice while doing MBW, although spirometry is also done with an animated game concomitantly.

Aside from differences in duration and mode of entertainment, spirometry can be difficult given the maneuvers of forced inspiration and expiration required, especially for those doing the test for the first few times. MBW, on the other hand, requires only passive cooperation and is therefore easily feasible during infancy and early childhood [1]. Of note, one participant in our study preferred spirometry over MBW because of oral bleeding that occurred during MBW due to dental wiring.

While MBW was preferred over spirometry, we acknowledge that it is challenging for the technician performing MBW to successfully encourage children to stay still during each trial. This is essential to avoid leaks. At the same time, sialorrhea was identified as a significant concern for almost all participants.

Many challenges faced our study. This was a single-center study with a limited number of participants with relatively rare pathologies. Furthermore, in comparison with CF patients who are known to have ongoing inflammatory and immunological processes, with more frequent follow-up visits during the year, and where LCI is proven to be sensitive in detecting early airway changes, our participants were not clinically as severely ill and did not have follow-up visits as frequently (three to four times per year). This was thought to be a limitation in detecting the sensitivity of LCI in this population. Moreover, LCI might be elevated in patients with PCD even in the absence of bronchiectasis. Lastly, the absence of an adapted radiological score for bronchiectasis evaluation in the pediatric population was another limitation, and modifications were applied to the Reiff score in order to use it in our study. This score was also not always applied to recently performed CT scans due to ethical and medical considerations preventing us from implementing CT and its associated radiation exposure to children as part of our study.

## 5. Conclusions

This study is one of only a few that have been conducted to investigate MBW in children with PCD-BE and may be the first to study MBW in children with nCFnPCD-BE. It shows that LCI, despite its longer duration and other challenges, might be a promising tool for detecting peripheral airway changes earlier than conventional spirometry or even detecting changes when spirometry fails to do so. While it has been shown that LCI is elevated in adult patients with nCFnPCD-BE, further studies are needed in children. This pilot study needs to be continued with a larger patient population and repeated measures of LCI in order to further study the role of MBW in the follow-up of children with PCD-BE and nCFnPCD-BE and to provide further comparison of MBW with spirometry and CT modalities in the assessment of lung function and disease progression in pediatric structural lung diseases.

## Figures and Tables

**Table 1:** Summary of demographic features of the study population.

	Total (*n* = 20)	PCD-BE (*n* = 9)	nCFnPCD-BE (*n* = 11)
Median age, years (range)	12 (5–18)	12 (8–12)	12 (5–18)
Sex, Male/Female	11/9	4/5	7/4
Male, %	55	44.4	63.6
Ethnicity (%)			
Caucasian	18 (90)	7 (77.8)	11 (100)
Person of color	2 (10)	2 (22.2)	0
Median BMI z-score (range)	0.7 (−1.3–2.3)	0.7 (−0.6–1.6)	0.6 (−1.3–2.3)

Abbreviation: *n* = number; BMI = body mass index; PCD-BE = primary ciliary disease bronchiectasis; nCFnPCD-BE = noncystic fibrosis, non-primary ciliary disease bronchiectasis.

**Table 2:** Values of LCI, spirometric parameters, and the modified Reiff score.

	Total (*n* = 20)	PCD-BE (*n* = 9)	nCFnPCD-BE (*n* = 11)
Median LCI (range)	7 (5–9.1)	7.3 (6.4–7.8)	6.8 (5–9.1)
Abnormal LCI (%)	9 (55) *	6 (66.7)	3 (27.3)
Median FEV1 z-score (range)	−0.6 (−2.4–1.3)	−0.5 (−1.2–0.2)	−0.8 (−2.4–1.3)
Abnormal FEV1 z-score (%)	2 (10)	0	2 (18.2)
Median FVC z-score (range)	0 (−1.9–1.5)	0.04 (−0.6–0.9)	−0.5 (−1.9–1.5)
Abnormal FVC z-score (%)	1 (5)	0	1 (9.1)
Median FEF 25–75% z-score (range)	−0.9 (−2.9–−0.1)	−0.9 (−2.1–−0.1)	−0.8 (−2.9–0.3)
Abnormal FEF 25–75% z-score (%)	3 (15)	2 (22.2)	1 (9.1)
Median modified Reiff score (range)	3 (1–7)	3.5 (1–7)	2 (1–7)

* Among the patients with abnormal LCI, the median index was 7.4, with a range between 7.2 and 9.1. Abbreviations: *n* = number; LCI = lung clearance index; FEV1 = forced expiratory volume in one second; FVC = forced vital capacity; FEF 25–75% = forced expiratory flow at 25–75%; PCD-BE = primary ciliary disease bronchiectasis; nCFnPCD-BE = non-cystic fibrosis, non-primary ciliary disease bronchiectasis.

**Table 3:** Associations between LCI and spirometric parameters.

	Normal LCI (%)	Abnormal LCI (%)	*p*-Value *
Normal FEV1 (%)	9 (45)	9 (45)	0.18
Abnormal FEV1 (%)	2 (10)	0 (0)	
Normal FVC (%)	10 (50)	9 (45)	0.36
Abnormal FVC (%)	1 (5)	0 (0)	
Normal FEF 25–75% (%)	9 (45)	8 (40)	0.66
Abnormal FEF 25–75% (%)	2 (10)	1 (5)	

* Associations between LCI and spirometric parameters. Abbreviations: LCI = lung clearance index; FEV1 = forced expiratory volume in one second; FVC = forced vital capacity; FEF 25–75% = forced expiratory flow at 25–75%.

## Data Availability

The data presented in this study are available on request from the corresponding author. The data are not publicly available in order to respect patients’ confidentiality.
